# Assessment Teams Experience of Barriers and Challenges When Assessing Loneliness and Depression Among Home‐Dwelling Elderly: A Qualitative Study

**DOI:** 10.1111/scs.70026

**Published:** 2025-04-16

**Authors:** Birgit Hauger, Randi Martinsen, Knut Hestad, Liv Skomakerstuen Ødbehr

**Affiliations:** ^1^ Department of Health and Nursing Sciences Inland Norway University of Applied Sciences, Faculty of Social and Health Sciences Elcerum Norway; ^2^ Department of Health and Nursing Sciences, and Inland Hospital Trust Inland Norway University of Applied Sciences, Faculty of Social and Health Sciences Elverum Norway

**Keywords:** care assessment teams, depression, elderly people, home care, home‐dwelling, loneliness, qualitative approach

## Abstract

**Background:**

Loneliness and depression in the elderly population are a worldwide challenge. Elderly people receiving home care are very vulnerable, as they often suffer from comorbidity and various other challenges.

**Aim:**

The study aim was to explore how care assessment teams experience assessing loneliness and symptoms of depression among home care recipients aged 65 years and older in planned home visits.

**Method:**

This study has a qualitative design. Data were collected during two phases. First, data from documents were collected in seven municipalities on 168 home care decisions made by assessment units during 1 month. Second, 10 caseworkers and 10 case managers from different care assessment teams in 20 municipalities located in four different regions in Norway were individually interviewed. Both data sources were analysed using qualitative content analysis.

**Results:**

Analysis of the documents revealed that only three of the 168 home care decisions described support for mental health. The analysis of the interviews resulted in three themes: (i) ‘Physical health has higher priority than mental health in home care’, (ii) ‘Being diagnosed is essential to receive support from home care’, (iii) ‘Major differences in mental health support between municipalities’.

**Conclusion:**

Caseworkers in care assessment teams find that many home‐dwelling elderly are struggling with loneliness and depression, but barriers on both organisational and administrative levels prevent them from providing appropriate interventions. Mental health is thus not systematically recorded in assessments, which may lead to loneliness and depression being overlooked. There are significant differences between municipalities regarding service offerings and follow‐up procedures for loneliness and depression, and both caseworkers and municipal leadership must ensure a system that provides comprehensive patient care.

## 1 Introduction

As many as 30%–50% of the adult population develop a mental disorder during their lifetime [1]. Common mental disorders are a broad term covering psychosocial disabilities and conditions associated with significant distress, impaired functioning or risk of self‐harm [2]. Such disorders often involve both mental and physical illnesses and may reduce life expectancy [3]. The World Health Organization (WHO) [2] emphasises that approximately 280 million people worldwide suffer from depression, indicating that mental health disorders are frequent [4]. Furthermore, the WHO reveals that loneliness and depression in elderly people are increasing worldwide [2, 5]. Loneliness is prevalent among elderly people, affecting about one in four in high‐income countries. Although severe loneliness is rare, its impact represents a significant public health and social challenge [6]. Loneliness has increased in recent years among the oldest people living alone [7]. Although loneliness is not classified as a pathological phenomenon [8], prolonged loneliness can increase the risk of institutionalisation and health problems [9]. Loneliness is commonly experienced in later life, although only a small proportion of the population is severely affected [6]. Loneliness is associated with stigma [10], and loneliness and limited social contact can have several negative health consequences, such as depressive symptoms, increased risk of dementia and premature mortality [11]. Loneliness is a subjective experience, characterised by a sense that something is missing in one's social relationships. Loneliness arises from a gap between actual and desired social connections. Theories explain this gap differently: A social loneliness perspective relates to unmet social needs, while the emotional discrepancy model highlights a mismatch between the ideal and the actual; thus a lack of attachment and closeness in social relationships. Loneliness can also result from predisposing factors and triggering events [8]. Severe emotional loneliness is often related to high levels of depression and stress, which can lead to a pathological condition [10]. Other consequences of loneliness are a reduced social network [12, 13], a higher risk of malnutrition due to reduced appetite [14], social isolation [15], long‐term and chronic disease [10, 16], poor self‐rated health [13, 16] and functional decline and death [17]. Loneliness is undesirable and should therefore be differentiated from the positive, self‐chosen aspects of being alone, also called solitude [18, 19].

Geriatric depression, occurring in individuals aged 65 years and older, is often accompanied by physical illness or cognitive dysfunction [20]. Common triggers include the loss of a spouse, relationships, health and social and professional life, especially among those living independently [21]. Symptoms resemble general depression, including depressed mood, loss of interest, decreased concentration, changes in weight, fatigue, agitation or retardation, sleep disturbances and/or suicidal thoughts [4]. Common consequences of geriatric depression are impaired functional capacity [20, 22, 23], reduced social participation [24], reduced quality of life [22, 23, 25], risk of suicide [22, 26] and increased mortality [20, 26]. These are all serious conditions in need of care and treatment by professionals.

One goal is for elderly people to manage their situation to enable them to live at home for as long as possible. Various factors influence the experience of good health, such as the sense of coping in life. The feeling that one's environment is predictable and manageable is vital for a sense of independence and autonomy [27]. The ability to use such personal resources is described by Antonovsky as the ‘sense of coherence’ (SOC). People's capacity to comprehend their situation and to find meaning in improving their health is vital for a feeling of empowerment and wellness [28].

Municipal care assessment teams, primarily composed of registered nurses (RNs), handle cases and administrative tasks, evaluate healthcare services and inform patients and home care nurses about entitlements. Diagnosing older individuals is the responsibility of general practitioners. Under the Health Personnel Act [29], municipal healthcare staff must provide comprehensive, preventative care for physical and mental health issues, while adhering to ethical guidelines [30].

Norwegian municipal healthcare services follow the ‘lowest effective level of care’ principle, meaning that the municipality first assesses whether the service can be provided at the lowest level and increases to higher levels of care if necessary. Lower levels of care are always a solution before more cost‐intensive interventions. This ensures that patients receive the best possible individually tailored care [31, 32].

Norwegian municipal healthcare services follow the ‘lowest effective level of care’ principle, meaning that the municipality first assesses whether the service can be provided at the lowest level and increases to higher levels of care if necessary. Lower levels of care are always a solution before more cost‐intensive interventions. This ensures that patients receive the best possible individually tailored care [33] and supports caring for elderly home‐dwellers [34, 35]. The interrelationship between loneliness and depression [36] calls for enhanced understanding of the needs of elderly individuals experiencing loneliness and depression. Additional research is essential to improve assessments during home visits and address patient needs effectively [35, 37, 38, 39].

## 2 Methods

### 2.1 Design

This study has a qualitative design. To explore the study aim, two data sources were employed to gain broader and deeper knowledge and understanding of how care assessment teams experience assessing loneliness and symptoms of depression among home care recipients aged 65 years and older in planned home visits. First, documentation of 168 home care decisions made by assessment units during 1 month were reviewed in seven municipalities. Second, to elicit nurses' experiences of interventions regarding loneliness and depression in the care of elderly home‐dwellers, individual interviews were conducted. 10 caseworkers and 10 case managers employed in 20 municipalities located in four regions of Norway were interviewed.

The Research Questions Are as Follows

1. What health interventions do care assessment teams assess for elderly home‐dwelling people who experience loneliness and have symptoms of depression?
2. What are the experiences of care assessment teams in assessing loneliness and symptoms of depression in elderly home care recipients?

### 2.2 Study Settings and Recruitment

Inclusion criteria for the documents were records from patients over 65 years old and care decisions made within 1 month.

The inclusion criteria for the interviews were caseworkers and case managers working in care assessment teams with at least 2 years' experience of conducting assessments during home visits.

#### 2.2.1 The Care Assessment Documents

The research aim was communicated to health leaders and care assessment team managers in the 30 municipalities invited to participate. Seven municipalities agreed to take part, and anonymous care decisions were collected from these in August–November 2022. Care assessment team managers extracted the written decisions from patient records generated over a month, providing anonymised written decisions to the research team, which stored the documentation digitally. The documents were reviewed for care decisions based on assessments describing care offered to patients.

#### 2.2.2 The Interviews

Invitations were sent to municipal leaders and care assessment team managers in 104 municipalities, explaining the project aim. Information was disseminated to caseworkers by their care assessment team managers and those who agreed to participate informed the research team by e‐mail. Twenty municipalities participated and telephone interviews were conducted from October 2022 to April 2023. Before the interviews, the informants signed consent forms and returned them to the research team. A semi‐structured interview guide was used [40], covering topics such as identifying and assessing loneliness and depression in elderly people during assessment visits. Examples of questions were: ‘How do you come to realize that elderly patients are lonely or depressed? How do you assess loneliness and symptoms of depression? What interventions are implemented for patients with loneliness and symptoms of depression’?

Interviews lasted 30–45 min, and were recorded and transcribed verbatim. There were 18 female and two male informants, averaging 45.5 years of age, primarily RNs. After 20 interviews, no new information emerged, and recruitment ceased.

### 2.3 Analysis

The documents and the interviews were analysed using qualitative content analysis [41]. Graneheim and Lundman's [41] approach distinguishes between manifest and latent content, where manifest refers to visible, obvious and literal meanings, while latent refers to underlying meanings. This process condensed the text while retaining its meaning, involving abstraction through codes, categories and subcategories, leading to interpretations and theme formulation [41]. Sufficient informants and care decisions were obtained, with no new information emerging after 20 interviews and analysis of 168 decisions [41].

#### 2.3.1 The Documents

All the 168 care decisions by the care assessment teams were reviewed several times to ensure that the information was captured and to gain a general overview. Subsequently, the text was systematised by simplifying and highlighting relevant information. The content was interpreted in order to understand the responses to specific questions about mental health (loneliness and depression) posed to the text, and care decisions concerning loneliness and depression were highlighted [41]. The analysis revealed few decisions on the assessment of home care interventions based on loneliness and depression. The documents relate to the manifest data, in line with the analysis process of Graneheim and Lundman [41].

#### 2.3.2 The Interviews

The transcribed interviews were read multiple times to gain an overall impression of the content. Based on the study aim, meaning units were identified and condensed into codes and subcategories while still preserving the core meaning [41]. Through reflection and discussion in the research team, the codes were interpreted and compared for differences and similarities, and the analysis revealed three themes representing the latent content. See Table 1 for an example of the inductive analysis process.

**TABLE 1:** Example of analysis process.

Meaning units	Condensed meaning unit	Code	Sub‐category	Theme
I think physical health has higher priority, even though we want to focus on mental health as well.	Physical health is more important than mental health. Physical health has higher priority than mental health.	Physical health has higher priority than mental health	Mental health is of lower priority	Physical health has higher priority than mental health in home care
Physical health is sort of more acute, for example when a patient with diabetes needs insulin.	Physical health seems more acute and urgent	Physical health problems seem to be more urgent than mental health problems	Nurses try to do their best to address mental health problems/struggles	
A diagnosis is the key to assessing needs for support and assistance for patients with loneliness and depression.	A diagnosis is the key to get support and assistance	A diagnosis is a governing factor	Without a diagnosis, the patients would not receive support and assistance	Being diagnosed is essential to receive support from home care
I believe there are considerable differences between small and large municipalities in services for elderly people and financial resources. There are probably also significant differences in how various assessment teams evaluate the mental health of elderly patients.	There are major differences between small and large municipalities in elderly care, finances, and how assessment teams evaluate elderly patients' mental health.	Major differences in municipalities in terms of healthcare provision and assessments of patients' health	Healthcare provision varies between municipalities	Major differences in mental health support between municipalities

### 2.4 Ethical Considerations

Permission to conduct the study was applied for to the Norwegian Agency for Shared Services in Education and Research (SIKT) [42] (No. 467880/2023). The Regional Committee for Medical and Health Research Ethics (REK) also approved the study (No. 478516/2023) because the researchers intended to obtain data from patients' medical records. Written information was provided concerning the study aim and data collection and storage.

The guidelines for risk and vulnerability analysis of the General Data Protection Regulation were followed. An electronic platform from the University of Oslo was used to collect, store and analyse data. This platform enables the collection of sensitive information for the Services for Sensitive Data system (TSD), which meets the strict legal requirements for digital processing and storage of sensitive research data [43].

In obtaining the 168 written care decisions, the research adhered to anonymity and privacy protection, following approval from the REK. Care assessment team managers printed anonymised care decisions, securely stored in a TSD database. During the interviews, the research was subject to voluntary, written informed consent, protection against harm, anonymity, the opportunity to review and withdraw from the study and secure data storage. Informants received written and oral information prior to the study and the consent form before participation [40, 44].

## 3 Results

### 3.1 The Documents

The analysis of the documents found that only three of the 168 decisions were suitable for home care interventions related to loneliness and depression. These were as follows: one decision stipulated weekly motivational conversations to improve the patient's mental health, another decision outlined weekly phone calls for addressing low mood and depressive thoughts, and the third described weekly support conversations to enhance self‐esteem. Additionally, two decisions mentioned symptoms of loneliness and depression, but no interventions were specified. It remained unclear whether some patients received other mental healthcare services due to incomplete descriptions in the decisions.

### 3.2 The Interviews

Three themes describe the experiences of municipal care assessment teams in assessing loneliness and symptoms of depression: (i) ‘Physical health has higher priority than mental health in home care’, (ii) ‘Being diagnosed is essential to receive support from home care’ and (iii) ‘Major differences in mental health support between municipalities’.

The findings are presented below, where statements are marked with CM (case manager) and CW (caseworker) and the number of the interview.

### 3.3 Physical Health Has Higher Priority Than Mental Health in Home Care

Although most informants mentioned that they focused on both physical and mental health during their assessment visits, they all still generally focused more on physical health. One said:

Physical health has higher priority than mental health, especially in older patients (…) Physical health is sort of more acute, for example when a patient with diabetes needs insulin. I'm doing my best, trying to ensure that the patients receive the best possible care. (CW1)

It seemed that physical health was easier to address and that physical health was prioritised even though it was also seen as desirable to consider mental health. There appears to be a less well‐established culture for assessing mental health in elderly patients.

Most informants reported lacking adequate tools to help them assess patients' mental health effectively, as one explained:

Without a suitable mental health assessment tool, we rely on observations and direct inquiries with patients. Clear, specific questions about mental health are essential, but patients often avoid answering properly and appear uncomfortable (…) Time constraints and busy schedules sometimes prevent us from considering the holistic perspective. (CW8)

The assessment tools are apparently not suitable for evaluating the patients' mental health. Increasing mental health‐related inquiries encourages patients to openly discuss their emotions, potentially helping more individuals to deal with loneliness and depression.

Despite closely monitoring all patient requirements, it was difficult to meet all their needs. The informants had acquired important observational skills through experience, but they needed more competence to make assessments that would result in optimal care for patients. Some stated that it was important to increase their skills in identifying problems. Training in how to conduct assessments seemed to be important to enhance the informants' confidence in interactions with patients.

### 3.4 Being Diagnosed Is Essential to Receive Support From Home Care

Several informants discussed the problem of diagnosis. One stated: ‘The ones without a diagnosis don't get much help. We don't provide help for symptoms’ (CM8). A diagnosis is key to assessing care needs for patients with loneliness and depression. Without a diagnosis, providing adequate services can be challenging.

The informants were concerned about patients' level of physical and mental functioning and stated that they had an assessment tool that consists of two questions: ‘What is important to you?’ and ‘What do you need now?’. These questions come close to addressing mental health in the assessment form, although they are open‐ended and somewhat vague.

Few municipalities initiated mental healthcare plans for elderly patients with loneliness and depression. One informant explained:

‘We use the principle about starting with the lowest effective level of care the municipality can provide, which is daycare centres. To receive more mental healthcare, patients need a diagnosis. Without a diagnosis, it's difficult to access help.’ (CM4).

Several informants noted a gap between patients' needs and available municipal interventions, leaving some without mental health support. Caseworkers found there was little flexibility to assign mental health interventions and disagreed with the low priority of mental health care for elderly patients. This made it difficult to provide adequate mental health services to elderly home care recipients. In this study, elderly individuals were mainly offered low‐cost activities in daycare centres, although many needed more personalised care to prevent loneliness and depression.

### 3.5 Major Differences in Mental Health Support Between Municipalities

Municipalities varied greatly in their provision of interventions for lonely and depressed elderly home care recipients. One informant stated:

I believe there are considerable differences between small and large municipalities in services for elderly people and financial resources. There are probably also significant differences in how various assessment teams evaluate the mental health of elderly patients. (CW7)

Several informants reported providing various nursing interventions. However, one informant mentioned utilising home care services for elderly individuals with loneliness and depression: ‘Sometimes we provide home care to lonely and depressed elderly people if they have other health needs, but mostly they get no services from home care’. Another informant stated: ‘We make few decisions on mental health for older people because we try to organize things as low‐cost services’ (CM8).

Some municipalities offered transport to the daycare centre, but not all. Many informants felt that social contexts were important for preventing loneliness and depression, yet it was not always straightforward to encourage elderly people to attend. Insufficient support would prevent them from attending the daycare centre. A greater focus on transport would enable more patients to access the daycare centre, thus alleviating their loneliness.

Disagreements often arise between home care and mental health services about caring for patients with loneliness and depressive symptoms, which affects collaboration due to elderly people's reluctance to address mental health concerns. One informant declared: ‘Home care often replies: “No, we can't do anything about that, it's someone else's responsibility”’ (CM2).

Municipal management should clearly specify which teams are responsible for providing care to elderly patients with loneliness and depression. Inadequate collaboration between municipal care services frequently led to patients receiving little support in this area.

## 4 Discussion

Analysis of the documents and the interviews revealed that municipal care assessment teams find limited focus on mental health or assessment of loneliness and symptoms of depression. Elderly people with loneliness and depression often receive inadequate interventions for these issues and are mainly encouraged to attend daycare centres without personalised care.

Furthermore, the absence of a particular mental health assessment form may result in inconsistent evaluations. This study indicates that mental health has a lower priority than physical health, and the availability of healthcare services depends upon the organisation of services and the municipality concerned. Disagreements within municipal health departments may arise regarding responsibility for providing care to older patients with mental health issues.

### 4.1 Limited Focus on Mental Health

The results revealed that home care mainly focuses on physical health, which concurs with previous research [36]. This may make care workers overlook psychosocial needs and neglect the overall perspective for patients who often have complex and multifaceted conditions [45]. The limited assessment of mental health needs prevents care assessment teams from providing comprehensive assessments. This contradicts Aaron Antonovsky's theory of salutogenesis, where research indicates that SOC is strongly related to health, especially mental health, and that the SOC seems to be a health‐promoting resource [28].

The informants also highlighted the challenge of obvious disparities in mental health support between municipalities, such as access to transport to daycare centres. Nonetheless, all municipalities were obliged to assess interventions according to the ‘lowest effective level of care’ principle, as recommended by the Norwegian Directorate of Health [46]. The findings clearly revealed that the elderly patients rarely received help from home care or mental healthcare for symptoms of loneliness and depression, but were often offered to join a daycare centre to meet other patients and improve social contact. Furthermore, the informants were upset at their inability to offer patients more individualised interventions, even though daycare centres were intended to fulfil patients' needs for social interaction. Research reveals the importance of attending daycare centres to complement other necessary healthcare and support [47], and that elderly people using a daycare centre have better physical and mental health than those receiving only home care [48]. This is in line with salutogenic theory, where health‐promoting interventions are essential for coping and for finding meaning in life [28]. However, research presents somewhat varied findings on whether daycare centres for older people mitigate loneliness and depressive symptoms [49, 50]. This suggests that home care that includes a preventative mental health approach might be adequate for elderly patients in combination with a daycare centre. However, it is essential to ask patients directly about their needs and enable them to participate in their own health management, which also implies acting from a salutogenic perspective [28].

### 4.2 Arbitrary Recording of Mental Health

The findings reveal no explicit questions about mental health in the assessment form, making it difficult for the informants to provide comprehensive and holistic care. This means that caseworkers conducting assessment visits need to rely more on their own experience and expertise to determine the mental health status of elderly patients. Home care nurses have the competence to observe loneliness and depression, but if this is not part of the documentation system, it may not be followed up, resulting in undetected loneliness and depression symptoms and thus inadequate assessment of mental health, as also stated in research [36, 51]. Importantly, a salutogenic perspective emphasises mental health and coping through the relationship between the patient and the nurse. This is also shown in research showing that nurse–patient interaction is a significant health‐promoting resource for the quality of life of elderly people [52]. Good nurse–patient relationships establish a sense of care, and listening to patients talk about their lives helps nurses to identify and assess symptoms of loneliness and depression. This does not necessarily require assessment forms, but it does require time and knowledge of mental health and SOC. An awareness of a salutogenic approach to health promotion would be of great benefit in practice to enhance the assessment of mental health issues in elderly home care recipients [28].

This study revealed that the informants were interested in considering the mental health nursing perspective of these patients, in line with professional ethical guidelines [30]. They made efforts within their available resources, although implementing new assessment procedures and interventions is a systemic responsibility. An important factor is that many elderly people find it difficult to talk about their mental health [53], which can increase the difficulty for care assessment teams to obtain an accurate picture of patients' mental health. A well‐designed assessment form would be superior to random assessment [54]. The lack of specific assessment questions on mental health issues might explain why few care decisions are made for patients with loneliness and depression. To ensure more accurate assessments of mental health and reduce the risk of errors and underestimations, specific questions on mental health should be included, as suggested in recent research [13, 36], but it is also important for nurses to utilise their expertise to assess mental health. This is in line with legislation [55, 56] and professional ethical guidelines [30]. Early identification of elderly individuals who are struggling followed by appropriate interventions based on the SOC approach can prevent mental health problems [27]. It is important to note that an assessment form cannot replace human contact. A nurse's observational skills, assessment competence and relationship with patients have a significant effect on how patients open up about their problems. Improving the screening tool is not enough—each nurse's attitude, knowledge and clinical competence are crucial for the quality of care.

In line with recent research, it is important to develop competence in systematic observation, identification of mental health problems, assessment, and care provision for elderly home‐dwellers [34]. This should enhance social participation and enable these elderly people to live at home for as long as possible, which is also important for their mental well‐being [57]. Early observation of symptoms and correct assessment and information can help to ensure comprehensive healthcare and support [13, 39, 56, 58]. The current organisation of health care is far from optimal to capture loneliness and depression in elderly home care recipients. It is crucial to further develop healthcare services to ensure the provision of high‐quality mental health care to this group.

### 4.3 Limitations in the Healthcare System

The findings revealed that a diagnosis is the key to healthcare interventions, and that care assessment teams are generally more concerned with functioning and needs than with diagnoses. This may constitute a knowledge gap that prevents teams from making adequate assessments of services needed. This could imply a stereotypical perception of elderly patients' needs that is not grounded in the identification of issues and interaction between healthcare providers and patients [59]. Loneliness is not a diagnosis, but a condition that can lead to pathology, with important consequences for the patient. Addressing loneliness can prevent illness and be cost‐effective over time. It is therefore crucial to prevent and address mental health issues and enhance meaningfulness in patients' lives to enable them to live at home for as long as possible, aligning with a salutogenic perspective [27, 28] and policy directives [35].

Formal collaboration on comprehensive care assessment based on ethical guidelines seems to be lacking [30]. Mental health illnesses in elderly people have low status among clinicians due to time constraints, rigid policies, staff shortages, poor continuity and stringent documentation requirements. There are thus many obstacles to providing care to elderly individuals with mental health problems [60], which concurs with the findings of the present study of disagreements about who should provide care to older people with symptoms of loneliness and depression. Neither home nursing nor mental healthcare met these patients' mental health needs, confirming their low priority. In line with Norwegian legislation and several reports and recommendations, age should not be an obstacle to assessment of necessary healthcare [29, 34, 55, 56, 61]. However, as emphasised by the Office of the Auditor General [58], elderly people with mental health symptoms do not receive the care they need due to limited understanding and competence of clinicians and may consequently be passed from one agency to another. It is problematic that patients become pawns in a system where neither home nursing nor mental health services can offer adequate support and assistance. This does not promote health and contradicts the fundamental principles of a salutogenic perspective. Instead, it raises a management issue regarding how the municipality can address this in a holistic manner and strive for best practice in accordance with legislation [55].

This study demonstrates that home care primarily prioritises physical health interventions, despite the informants' desire to provide holistic care. Limitations of the system prevent care assessment teams from addressing loneliness and depression due to inadequate interventions and low priority for these patients, which conflicts with ethical guidelines [30] and holistic nursing care principles. Financial and human resource constraints in municipal health care and limited time and knowledge compromise service quality [36, 62, 63], with considerable negative effects on elderly home care recipients. To facilitate prolonged independent living for elderly individuals, municipal care services must enhance their efforts to establish support systems [35] and implement interventions to alleviate loneliness and depression, thus averting adverse outcomes. Adequate municipal mental healthcare is deemed essential for the elderly population [64], and more effective assessments may free up resources for additional service improvements [65].

### 4.4 Strengths and Limitations

To achieve credibility and transferability, the authors attempted to describe the entire study and present quotes that represented the informants' voices. Dependability was attempted by following the steps in the analysis of Graneheim and Lundeman [41]. The research team discussed their preunderstandings to prevent them from guiding the interviews. The entire research team was involved in the analysis process and the development of all sections of the article [41, 66]. Reflexivity was discussed throughout the research process, taking account of preconceptions, personal preferences and the concepts and theories used in the manuscript to enhance credibility. Nonetheless, the authors' preconceptions and preferences may still have influenced the manuscript [40].

Telephone interviews provide efficient and accessible data collection across diverse geographical areas, ensuring participant anonymity [67]. They reached respondents from across urban and rural Norway. However, the absence of nonverbal cues in telephone interviews may have prevented the capture of crucial information. While the telephone interviews generated ample data, face‐to‐face interviews could have offered additional insights.

The study was conducted in the Norwegian context, with the potential for transferability to countries with a similar organisation of health care. Conducting the study in a Norwegian setting was beneficial in raising awareness among care assessment teams about the importance of not overlooking mental health in elderly patients.

### 4.5 Implications for Practice and Further Research

Care assessment teams need to have a salutogenic perspective, while attitudes, knowledge and clinical insight are important to improve assessments. It is crucial to address the lack of focus on mental health in assessment tools to enhance healthcare for this patient group, and to raise awareness of prioritising patients' mental health. Research indicates that the assessment of elderly people with loneliness and depression is often overlooked in home care, with a predominant focus on physical health and the requirement of a diagnosis to access adequate support. This discrepancy leads to significant disparities between municipalities. Further research is needed to develop a national assessment framework and improve preventative interventions, enabling elderly patients to stay at home longer, aligning with a salutogenic perspective [27] and national reform goals [35].

## 5 Conclusion

Caseworkers in care assessment teams find that many home‐dwelling elderly are struggling with loneliness and depression, but barriers on both organisational and administrative levels prevent them from providing appropriate interventions. Mental health is thus not systematically recorded in assessments, which may lead to loneliness and depression being overlooked. There are significant differences between municipalities regarding service offerings and follow‐up procedures for loneliness and depression, and both caseworkers and municipal leadership must ensure a system that provides comprehensive patient care.

## Author Contributions

B.H., R.M., K.H. and L.S.Ø. contributed to the study design. B.H. performed data collection. B.H., R.M. and L.S.Ø. performed the data analysis with inputs from K.H. B.H., R.M., K.H. and L.S.Ø. drafted the manuscript. All authors performed critical reviews and approved the final version.

## Ethics Statement

Norwegian Agency for Shared Services in Education and Research (SIKT) has approved the study (No 467880/2023). The Regional Committee for Medical and Health Research Ethics (REK) has approved the study (No 478516/2023). All informants provided written informed consent before participating in the study.

## Conflicts of Interest

The authors declare no conflicts of interest.

## Data Availability

The data that support the findings of this study are available on request from the corresponding author. The data are not publicly available due to privacy or ethical restrictions.
